# The Development of a Stereo Vision System to Study the Nutation Movement of Climbing Plants

**DOI:** 10.3390/s24030747

**Published:** 2024-01-24

**Authors:** Diego Rubén Ruiz-Melero, Aditya Ponkshe, Paco Calvo, Ginés García-Mateos

**Affiliations:** 1 Computer Science and Systems Department, University of Murcia, 30100 Murcia, Spain; diegoruben.ruiz@um.es; 2 Minimal Intelligence Laboratory (MINT Lab), University of Murcia, 30100 Murcia, Spain; ponkshe.aditya@gmail.com (A.P.); fjcalvo@um.es (P.C.); 3 Department of Philosophy, University of Murcia, 30100 Murcia, Spain

**Keywords:** plant nutation movement, computer vision, image processing, climbing plants

## Abstract

Climbing plants, such as common beans (*Phaseolus vulgaris* L.), exhibit complex motion patterns that have long captivated researchers. In this study, we introduce a stereo vision machine system for the in-depth analysis of the movement of climbing plants, using image processing and computer vision. Our approach involves two synchronized cameras, one lateral to the plant and the other overhead, enabling the simultaneous 2D position tracking of the plant tip. These data are then leveraged to reconstruct the 3D position of the tip. Furthermore, we investigate the impact of external factors, particularly the presence of support structures, on plant movement dynamics. The proposed method is able to extract the position of the tip in 86–98% of cases, achieving an average reprojection error below 4 px, which means an approximate error in the 3D localization of about 0.5 cm. Our method makes it possible to analyze how the plant nutation responds to its environment, offering insights into the interplay between climbing plants and their surroundings.

## 1. Introduction

The nutational movement of climbing plants refers to the rhythmic, circular, or nodding motion exhibited by certain plant parts, such as stems, tendrils, or growing tips, as they explore and interact with their environment during the process of climbing [1]. This movement is often associated with the search for support structures, such as poles, trellises, or other plants, which the climbing plant can use for stability and upward growth [2]. It occurs as a result of differential growth rates on different sides of the plant organ. As the plant grows, the cells on one side of the organ elongate more rapidly than those on the opposite side, causing the organ to bend or curve [3]. This bending or curving allows the plant to explore its surroundings and find a suitable support for climbing.

Climbing plants, such as common beans (*Phaseolus vulgaris* L.), employ various mechanisms for climbing, including twining, where the plant winds around a support, and the use of tendrils, specialized structures that coil around objects for support [4].

The study of nutation movements in climbing plants is part of the broader field of plant tropisms, which involves the growth responses of plants to external stimuli such as light, gravity, and mechanical touch, first studied in the pioneering works of Charles Darwin [5]. This nutation movement allows climbing plants to optimize their growth in response to their environment, increasing their chances of successful climbing and their access to sunlight for photosynthesis. Moreover, it has been the inspiration for the development of new techniques and methods in robotics [6], computational intelligence [7,8], and other bioinspired innovations [9].

This current study of nutation and other plant movements requires the recording of time-lapse images of plants, due to the long time span in which the movements occur, and the use of artificial vision algorithms for the efficient analysis of the videos obtained. For example, Navarro et al. [10] developed a specific capture chamber to monitor the growth of plants at intervals from 30 s to hours or even days. The captured images were then analyzed with computer vision algorithms based on thresholding, morphological operators, and blobs detection. Using the parameters obtained from the blobs, features such as area, length, compactness, and perimeter were extracted for further analysis. Stolarz et al. [11] developed software called the “Circumnutation Tracker” to analyze the circular movement of the tip of the plants in zenithal time-lapse videos. The X and Y positions of the tip are manually selected by the user, and the system includes useful tools for the visualization and characterization of the plant movement, automatically extracting circumnutation parameters.

More recently, Tenzer and Clifford [12] proposed a technique to analyze the growing movement of hydroponic plants using different neural networks on grayscale images. U-net, Linknet, FPN, PSPNet, and a 34-layer ResNet architecture were used for comparison, obtaining a maximum area under the curve (AUC) for the validation set over 0.92. Another recent method based on deep learning was presented by Mao et al. [13], which proposed a U-net network architecture to segment the plant. This method is an improvement of their free software “Plant Tracer”, which analyzed plant movement using the classic computer vision techniques of object tracking and blob detection. Díaz-Galián et al. [14] presented a methodology to analyze the movement of plants using different reference points, which were obtained from image analysis. This methodology included coordinates transformation, curve fitting, and statistical analysis, allowing a comparison between different plant species. Plant movement has also been analyzed using other types of sensors. For example, Geldhof et al. [15] developed a digital inertial measurement unit (IMU) sensor to measure in real-time the movement of leaves, achieving a precision of pitch and roll angles under 0.5°. However, the exact 3D position of the leaves was not obtained.

The aim of this work is to develop a stereo system for the visual tracking of plant nutation movement, which enables obtaining the precise 3D location of the tip of the plant. This work was focused on the study of the common bean (*Phaseolus vulgaris* L.), since the ultimate purpose is to analyze the differences in the movement between using or not using a support pole for plants. Previous work from the MINT Lab research group suggests that the dynamic patterns of plant nutation are influenced by the presence of support to climb in their vicinity [2]. This fact has also been observed by other researchers. For example, Guerra et al. [16] analyzed the 3D kinematics of the movement of climbing plants, demonstrating that plants are not only able to perceive their environment but can also scale the movement of their tendrils depending on the size of the support.

The objective of this present study is not to analyze these kinematic changes under different environmental conditions but to develop computational tools for the accurate 3D measurement of plant tip movement that will be used in further studies. Consequently, for our analysis to be effective in a broader range of conditions, we obtained data on plants from different settings, i.e., with and without a support to climb onto. Since the proposed method is able to reconstruct the 3D position of the tip in both conditions, our method promises to offer a framework for future studies to analyze how plant nutation responds to different environments.

## 2. Materials and Methods

**Figure 1:**
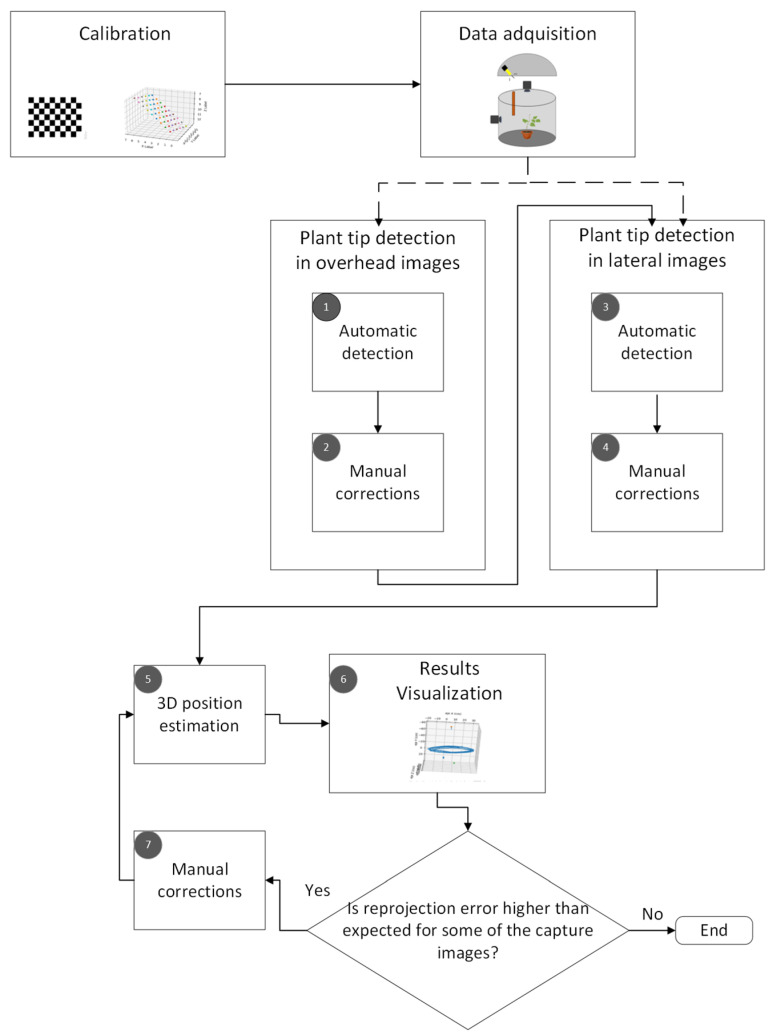
Pipeline for the proposed computer vision method used to track the plant tip movement.

The proposed computer vision method to track the movement of the plant tip is shown in Figure 1. It consists of five main stages: calibration, data acquisition, plant tip detection in the top and side images, 3D position estimation, and visualization of the results.

In the following subsections, the characteristics of the experimental setup and the stereo imaging system are presented first. Then, a brief introduction to the mathematical foundations and artificial vision algorithms used is given. Finally, the procedure used to estimate the position of the plant tip at each of the moments captured in the time-lapse videos is described.

### 2.1. Experimental Setup

The experiments were carried out by the MINT Lab research group at the Scientific and Technical Research Area of the University of Murcia (Spain). These experiments were conducted under controlled conditions and consisted of two scenarios. In the first scenario, the climbing plant was placed near a pole, which served as a support for the plant to grip when it reached its position; in the second scenario, the plant was placed within a cabin with no pole in it. The pole was 90 cm in height and 1.8 cm in diameter and was placed at a distance of 30 cm from the plant center. Concerning the soil, a mixture of peat moss and perlite (70–30%) was used throughout all the experiments. Both cabins were equipped with white parabolic reflectors to provide symmetrical lighting. The temperature of the growth chamber was kept constant at 20 °C and the relative humidity at 85% ± 5%. We have to note that, although this is a high relative humidity, plants are able to accomplish their life cycles successfully with no harm, especially in controlled conditions in which heat stress can be prevented. A L16:D8 h photoperiod was provided via high-pressure sodium lamps (Lumatek pulse-start HPS Lamp 250 W; height: 150 cm and photon fluence rate: 430 ± 50 μmol m^−2^ s^−1^ at leaf level). During the 8 h of darkness, a dim phototropically inactive green safelight (fluence rate under 5 μmol m^−2^ s^−1^) was activated, which was enough to take pictures in the dark using a 4.2 μm high dynamic range (115 dB) image sensor.

**Figure 2:**
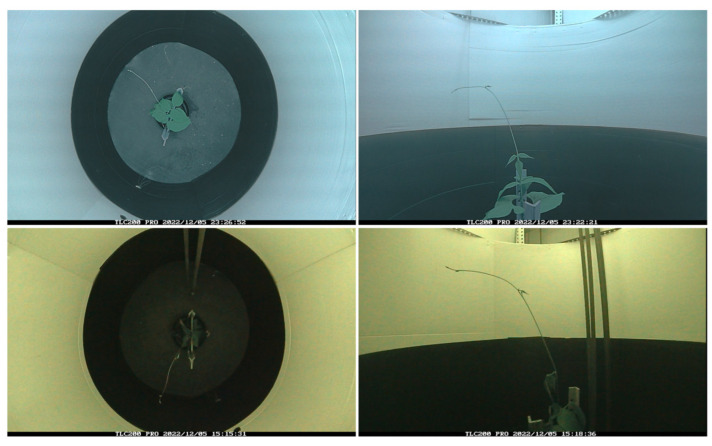
Images of the experiments conducted. At the top, zenithal and lateral images of the experiment in the no-pole condition; at the bottom, images of the pole condition.

Figure 2 shows an example of these two scenarios, with and without a supporting pole. Specifically, the plant species used for this experiment were the common bean (*Phaseolus vulgaris* L.). Seeds of the cultivar “Garrafal Oro Vega” variety were provided by Semillas Ramiro Arnedo S.A., Spain (https://www.ramiroarnedo.com). “Garrafal Oro Vega” is a variety with abundant foliage, having medium-sized leaves and containing 18–20 cm long pods. The plants went through multiple stages from germination to the start of the recording. The seeds took 48 h to germinate. Upon germination, the young seedlings were transferred to coconut fiber growing pellets and kept on propagation trays for further development. Once the first true leaves matured, which took around 8–12 days from the young seedling stage, healthy-looking, 25–30 cm tall plants were selected and transferred into the recording booths for video capture. The videos were recorded until the plant either grabbed the pole, or the tip came out of the experimental area (i.e., the area visible to the cameras), which usually happened after 3 or 4 days.

It can be observed in Figure 2 that a dark color was used at the bottom part of the booth. The purpose of this dark cover was to increase the contrast with the plant contour, thus enhancing the performance of the algorithm for extracting the position of the plant tip in the videos corresponding to the top view in time-lapse. We made sure that the white part remained disproportionately more than the black part. The proportion of the lower black part was calibrated so that the plants did not show any shade avoidance response, thereby altering the nutation movement.

Once the young seedlings were transferred to the coconut fiber growing pellets and kept on propagation trays for further development, 30 mL of water was initially added every day. Afterwards, when the plants started to mature, they were watered so that around 80% moisture was maintained throughout the growing stage. For recording purposes, the plants were transferred into big, black plastic pots. At the onset of the recording, 400 mL of water was added to each plant, which was found sufficient to keep the soil moist throughout the recording phase. No extra water was added during the four days of recording.

**Figure 3:**
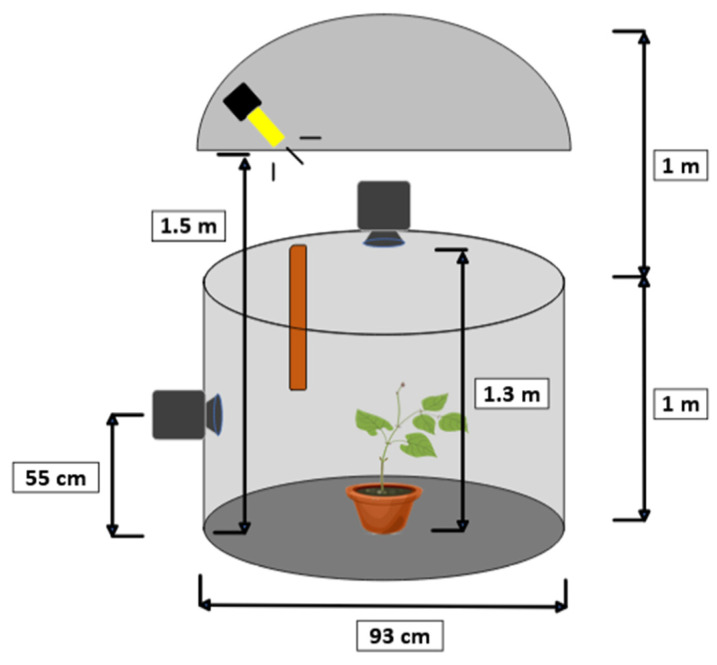
Configuration of the stereo imaging system that was used. The recording cabin was a cylinder of 1 m height and 93 cm radius. The lateral camera was situated at 55 cm from the floor, the zenithal camera at 1.3 m, and the lamp at 1.5 m. The upper reflector had a height of 1 m.

To estimate the nutational movement of the plant, a stereo pair of cameras was used, recording synchronously and in time-lapse. The target of the motion tracking was the plant tip. The configuration of the stereo vision system is shown in Figure 3. This imaging system consisted of two Brinno TLC200 Pro cameras (Brinno Ltd., Taipei, Taiwan) positioned at the top and side of the plant, forming a 90° angle. The technical specifications of the cameras are shown in Table 1. This top-side configuration of the cameras was chosen because it can offer high accuracy in detecting the tip of the plant, although it may not be the best choice for tracking other parts of the plant.

To synchronize the captured images, the recording was started simultaneously on both cameras. Each camera used its own internal clock. The images were captured at one-minute intervals for a continuous 24 h recording, lasting from 3 to 4 days. Because each camera had its own clock, there could be a slight offset in the images between the two cameras for some recordings. To determine the number of frames corresponding to this offset, the light–dark cycle used in the experiments to provide the L16:D8 photoperiod was used. For this purpose, the zenithal images were used as a reference, based on the photoperiod changes. During one of the lighting changes, the offset between the top and side view images was computed, measured as the number of frames of difference. This number was used in the further analysis of the sequence.

### 2.2. Mathematical Methods

In general, a camera maps 3D points in space to 2D points on the image plane. The cameras used in the experiments were modeled using a pinhole camera model. In a pinhole model, a ray from a point in space passes through a fixed point called the projection center. The intersection of this ray with a chosen plane in space, the image plane, is the projection of the point on the image plane [17]. The projective transformation given by a pinhole camera model is as follows:

(1) $$sp\;=\;A\left[Rt\right]P_{w}$$

where:

- *s* is an arbitrary scaling factor of the projective transformation.
- p is the 2D pixel on the image plane.
- $P_{w}$ is the 3D point expressed in the world coordinate system.
- A is the matrix with the intrinsic parameters of the camera.
- R and t are the rotation and translation, respectively, describing the coordinate change from the world to the camera coordinate system.

The matrix with the intrinsic parameters is composed of the focal lengths, $f_{x}$ and $f_{y}$, expressed in pixel units, and the principal point of the camera, ($C_{x}$, $C_{y}$).

(2) $$A=\left[\begin{array}{ccc} f_{x} & 0 & C_{x}\\ 0 & f_{y} & C_{y}\\ 0 & 0 & 1 \end{array}\right]$$

This matrix with the intrinsic parameters does not depend on the scene, so it can be reused once estimated, as long as the focal length remains fixed. On the other hand, matrix [R|t] contains the extrinsic parameters of the camera. This matrix performs the homogeneous transformation and represents the change of basis from the world coordinate system to the camera coordinate system, as follows:

(3) $$\left[\begin{array}{c} X_{c}\\ Y_{c}\\ Z_{c}\\ 1 \end{array}\right]=\left[\begin{array}{cccc} r_{11} & r_{12} & r_{13} & r_{14}\\ r_{21} & r_{22} & r_{23} & r_{24}\\ r_{31} & r_{32} & r_{33} & r_{34}\\ 0 & 0 & 0 & 1 \end{array}\right]\left[\begin{array}{c} X_{w}\\ Y_{w}\\ Z_{w}\\ 1 \end{array}\right]$$

Combining these two matrices yields the projective transformation, which maps the 3D points in the world to the 2D points on the image plane in normalized camera coordinates.

(4) $$s\left[\begin{array}{c} u\\ v\\ 1 \end{array}\right]=\left[\begin{array}{ccc} f_{x} & 0 & C_{x}\\ 0 & f_{y} & C_{y}\\ 0 & 0 & 1 \end{array}\right]\;\left[\begin{array}{cccc} r_{11} & r_{12} & r_{13} & r_{14}\\ r_{21} & r_{22} & r_{23} & r_{24}\\ r_{31} & r_{32} & r_{33} & r_{34}\\ 0 & 0 & 0 & 1 \end{array}\right]\left[\begin{array}{c} X_{w}\\ Y_{w}\\ Z_{w}\\ 1 \end{array}\right]$$

The pinhole camera model does not consider the distortion caused by the lenses used in the cameras. To accurately represent a real camera, the camera model includes both radial and tangential lens distortion. Radial distortion occurs when light rays bend more at the edges of the lens than at the optical center. The radial distortion coefficients model this type of distortion as follows:

(5) $$xdistorted\;=x\left(1\;+\;k_{1}r^{2}\;+\;k_{2}r^{4}\;+\;\;k_{3}r^{6}\;\right)\;ydistorted\;=y\left(1\;+\;k_{1}r^{2}\;+\;k_{2}r^{4}\;+\;\;k_{3}r^{6}\;\right)$$

where:

- x, y is the location of the pixel without distortion.
- $k_{1}$ $k_{2}$, and $k_{3}$ are the radial distortion coefficients of the lens.
- $r^{2}=x^{2}+y^{2}$.

On the other hand, tangential distortion occurs when the lenses and the image plane are not perfectly parallel. In this case, the tangential distortion coefficients model this type of distortion as follows:

(6) $$xdistorted\;=x\;+\;\left[p_{2}\left(r^{2}+2x^{2}\right)+2p_{1}xy\right]\;ydistorted\;=y\;+\;\left[p_{1}\left(r^{2}+2y^{2}\right)+2p_{2}xy\right]$$

where:

- x, y is the location of the pixel without distortion.
- $p_{1}$ and $p_{2}$ are the tangential distortion coefficients of the camera.
- $r^{2}=x^{2}+y^{2}$.

Using this basis, the computer vision algorithms that have been used in the implementation of the pipeline for the method used to track the plant nutation movement are described in the following points.

#### 2.2.1. Perspective-n-Point (PnP)

The pose computation problem consists of solving the rotation and translation of an object with respect to the camera, while minimizing the reprojection error for correspondences between the 3D points in the world and the 2D points on the image. Several methods have been proposed to estimate the pose of an object relative to the camera [18,19]. These methods can be used for calibration using a planar calibration object [20].

#### 2.2.2. Calibration of a Stereo Pair System

The procedure outlined in the previous subsection enables the obtaining of the intrinsic parameters of the camera as well as the camera pose relative to the calibration object. If the calibration object is visible to both cameras, we have the pose $\left(R_{1},t_{1}\right)$ of the first camera relative to the calibration object and the pose $\left(R_{2},t_{2}\right)$ of the second camera. These two poses are related as follows:

(7) $$R_{2}=R\times R_{1}\;;\;t_{2}=R\times t_{1}+t$$

Therefore, by using the previous equation, it is possible to obtain the pose of the first camera relative to the second camera. Once the pose of the first camera relative to the second one is obtained, the essential matrix can be constructed as follows:

(8) $$E=\left[\begin{array}{ccc} 0 & {-t}_{2} & -t_{1}\\ t_{2} & 0 & {-t}_{0}\\ {-t}_{1} & t_{0} & 0 \end{array}\right]\times R$$

where $t_{i}$ are the components of the translation vector $t=[t_{0},t_{1},t_{2}$]. From this, the fundamental matrix can be computed as follows:

(9) $$F={CameraMatrix}^{-T}\times E\times {CameraMatrix}^{-1}$$

#### 2.2.3. Background Segmentation

The background segmentation problem involves detecting changes that occur in a sequence of images. Pixels in an image are classified either as part of the background or as part of an object of interest based on a background model. In real applications, depending on the characteristics of the problem, different methods have been proposed: models based on color thresholding, clustering, and fuzzy logic, and, more recently, neural network-based models [21] have been used. The usual steps in image segmentation are:

1. The background model is initialized with some of the video frames.
2. Once the background model is initialized, each pixel in the image is classified based on the background model.
3. The background model is updated with information from the last processed image.

In the algorithms developed to extract the position of the plant’s tip in time-lapse images, the Gaussian mixture method was used. This method uses multiple Gaussian functions per pixel to model the background of the scene. This method takes into account that a pixel can have different states over time. The implementation of this method in the OpenCV library is based on Zivkovic [22].

### 2.3. Motion Tracking and 3D Position Estimation

This section first describes the procedure used to calibrate the stereo system. Next, the steps in the process of extracting the position of the plant tip from the images captured by the stereo system cameras are outlined. Finally, it describes how the nutation motion of the plant was tracked based on the 2D position of the extracted plant tip from the images.

#### 2.3.1. Camera Calibration

The stereo system calibration was carried out following the procedure described in the previous subsection. The calibration process was conducted in two phases. First, each of the stereo cameras was individually calibrated. A calibration object with a 10 × 7 chessboard pattern and dimensions of 59.4 × 84.1 cm was used for the calibration. These dimensions were chosen to cover a large part of the image plane, allowing the precise estimation of the distortion coefficients.

Once the individual calibration of the cameras was completed, the stereo system calibration was performed to estimate the projection matrices for each camera and the fundamental matrix. In this case, a calibration object with dimensions of 42.0 × 59.4 cm was used, visible in both cameras for a sufficient number of poses to carry out the stereo system calibration. Table 2 shows the results of the calibration, measured in terms of the reprojection error, that is, the distance from the calibration pattern key points detected in the images to a corresponding world point projected into the same images.

**Figure 4:**
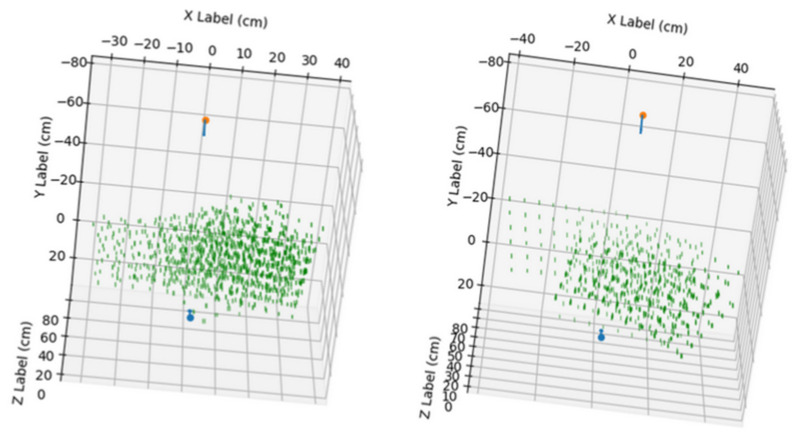
Points used in calibration of the stereo system (in green). **Left**: calibration scenario without pole. **Right**: calibration scenario with pole. The small red and blue arrows indicate the camera’s position in the scene.

In Figure 4, the points used in the calibration of the stereo system can be observed, combining the positions of all the images used for calibration.

#### 2.3.2. Detection of the Plant Tip in the Overhead View

**Figure 5:**
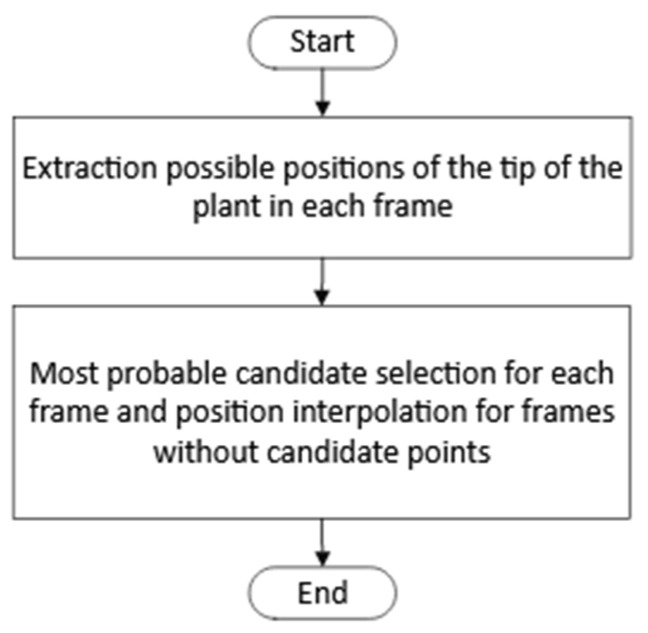
Procedure designed for extracting the plant tip position.

**Figure 6:**
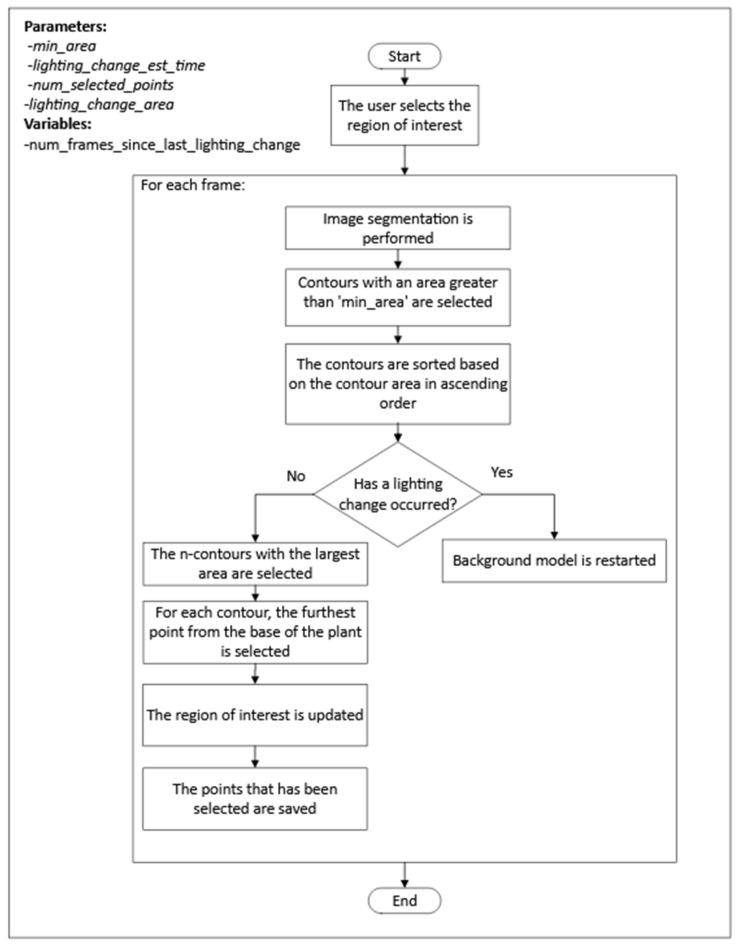
Algorithm for extracting possible positions of the plant tip in each frame of the top view.

The process consisted of two steps, as shown in Figure 5. First, for each frame, *n* points, where the plant tip could be located, were detected. Once the candidate points were obtained, a selection process for the position of the plant tip was performed for each frame, followed by interpolation for frames where no candidate points were detected. The steps of the first step can be seen in Figure 6.

The following are the highlights of the algorithm used in the first step of the process:

- The segmentation of the plant contour was performed using the mixture of Gaussians algorithm.
- After each change in lighting, the process reset the background model to reduce the stabilization time.
- To detect when a lighting change occurred, the process checked the area of the selected contours, and, if the area exceeded a threshold specified by the *lighting_change_area* parameter, it was assumed that a lighting change had occurred.
- After a lighting change, the process did not check again for a lighting change until after the frame period specified by the *lighting_change_est_time* parameter to prevent the repeated detection of a lighting change in every frame after the background model had been reset.

The process for selecting the plant tip position among the candidate points followed these two criteria:

1. If there was only one candidate point, it was selected.
2. If there was more than one candidate point, the point closest to the last point selected for previous frames was selected. In segments of frames where there was no candidate point, the position of the plant tip was estimated by performing linear interpolation using the plant tip position in the previous and subsequent frames of the segment.

#### 2.3.3. Detection of the Plant Tip in the Lateral View

**Figure 7:**
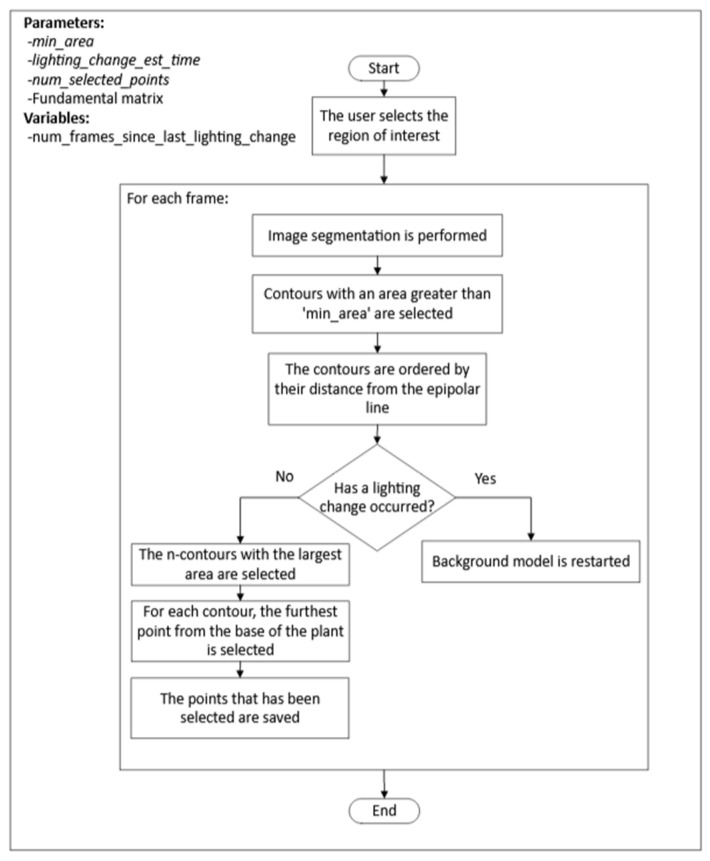
Algorithm for extracting possible positions of the plant tip in each frame of the side view.

This process consisted of two stages, as in the case of the process used for extracting the position of the plant tip in the top view images. First, for each frame, the *n* points, where the plant tip could be located, were detected. Once the candidate points were obtained, a selection process for the plant tip position was executed for each frame, followed by interpolation for frames where no candidate points were detected. The steps of the first stage are depicted in Figure 7.

The highlights of this algorithm used in the first stage of the process are:

- The segmentation of the plant contour was performed using the mixture of Gaussians algorithm, just as for the segmentation of the plant contour in the top view.
- For detecting changes in lighting, the same procedure used in processing images from the top view was followed.
- There were cases where multiple fragments of the plant contour are obtained when applying the background model to the image. In these cases, the use of the epipolar line helped to identify the fragment where the plant tip was located.
- Once the contours near the epipolar line were selected, a minimum path algorithm was used to select the potential position of the plant tip within the contour.

The process for selecting the plant tip position among the candidate points followed the same criteria as indicated in the previous section.

#### 2.3.4. Correcting Wrong Detections and Obtaining the 3D Positions of the Plant Tip

**Figure 8:**
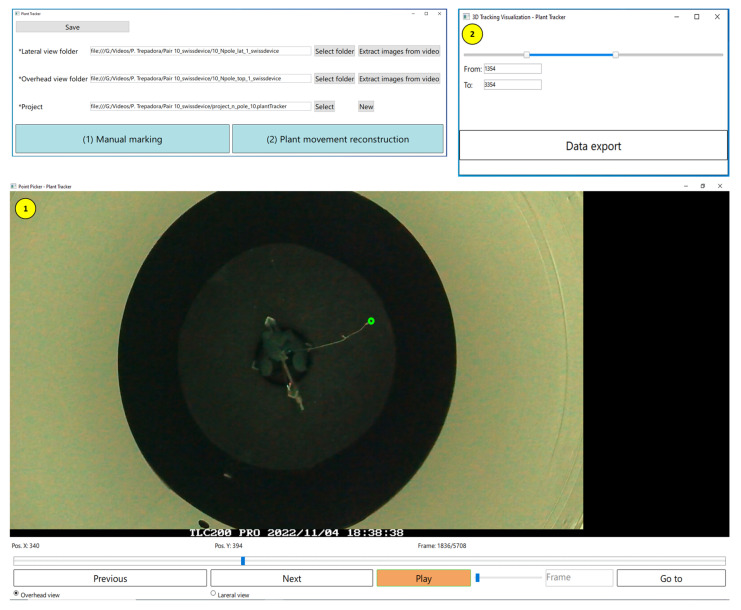
Sample view of the Plant Tracker tool developed to supervise the obtained locations. Top left: main window of the program; button “(1) Manual marking” opens the window labeled 1, and button “(2) Plant movement reconstruction” opens the window labeled 2.

The algorithms described in the previous subsections were not always able to detect the plant tip position in all cases, as mentioned in each section. For this reason, a “Plant Tracker” tool was developed to allow the user to modify the automatically performed plant tip extractions. This tool consists of three screens, as shown in Figure 8. Once the user has completed the manual corrections, the application allows the extraction of a CSV file with the estimation of the 3D position for a selected range of frames.

## 3. Results and Discussion

**Figure 9:**
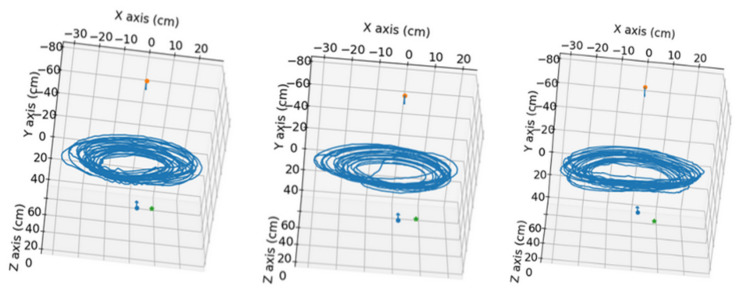
Visualization of the obtained nutation movement of the bean plants. **Left**: position result for video pair 4 without pole. **Middle**: position result for video pair 7 without pole. **Right**: position result for video pair 10 without pole. Red and blue arrows indicate the camera’s position in the scene, and the green dot marks the position of the pot’s base.

In this section, the results obtained in the processing of the available videos are presented. The videos were processed in pairs, corresponding to the zenithal and side views of each same experiment. The nutation movement followed by the tip of the bean plant in three of the conducted experiments is shown in Figure 9.

### 3.1. Results of the Detection of the Tip of the Plant

Table 3 shows the cumulative results of the type of detection used to extract the position of the plant tip in the frames of the time-lapse videos corresponding to the experiments. The types of detection correspond to the following categories: **Automatic:** The algorithm for extracting the plant tip position found only one candidate point. **Manual:** The points that the user had to correct. **Estimated:** The algorithm for extracting the plant tip position found more than one candidate point and selected the one that represented the most probable position of the plant tip. **Interpolated:** The algorithm for extracting the plant tip position did not find a candidate point. The plant tip position in the frame was determined by interpolating the position from the plant position in the preceding and subsequent frames.

**Figure 10:**
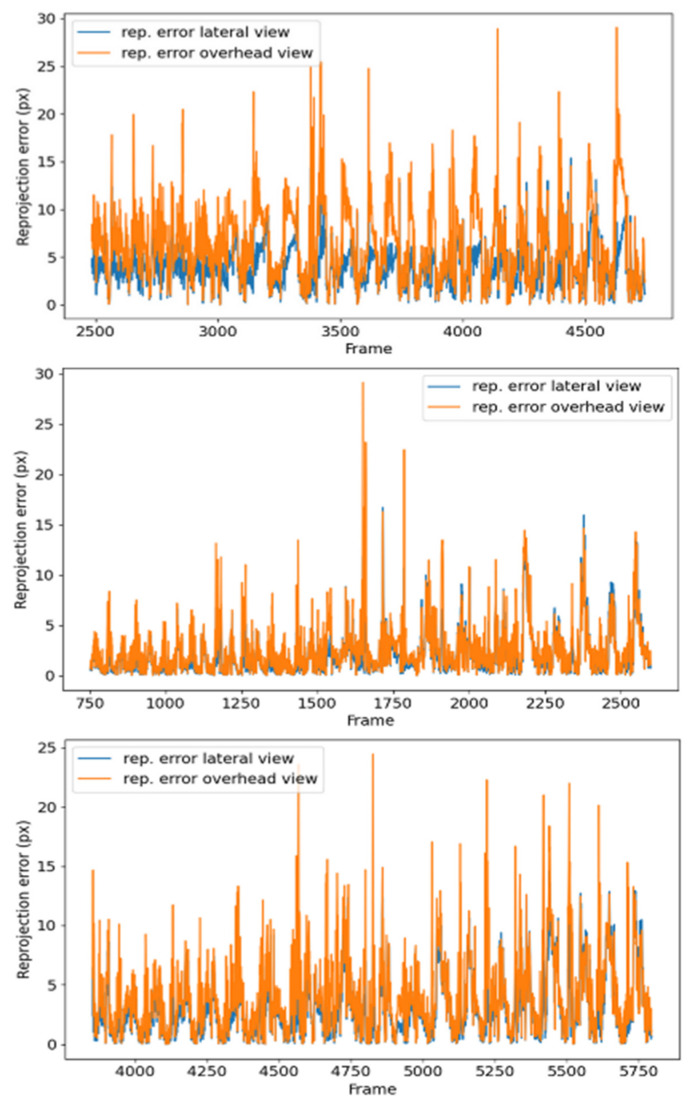
Visualization of reprojection errors of some videos along time. Top row: errors for video pair 4 without pole. Middle: errors for video pair 7 without pole. Bottom row: errors for video pair 10 without pole.

Figure 10 shows the reprojection error in pixels for each frame of the videos (side and top views) of the time-lapse within the range for which the 3D position estimation of the plant tip was performed.

### 3.2. Discussion of the Results

**Figure 11:**
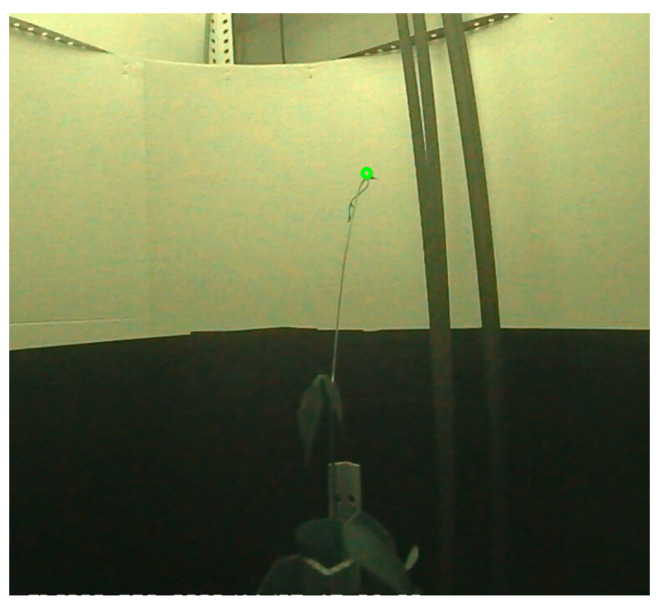
A sample of an incorrect detection of the plant tip in the side view image.

The plant tip detection algorithms were able to extract the correct position in a range of 86–98% of the cases, as observed in Table 3. The number of corrections made by the user was higher for the plant positions extracted in the lateral plant videos. This was mainly due to moments when the plant tip overlapped with the plant stem in the images. An example of this case is shown in Figure 11. This error is due to the use of a minimum path algorithm to find the plant tip in the segmented contour of the plant. When the plant tip overlapped with the stem, the position farthest from the plant’s base corresponded to the elevated part of the plant stem visible in the image.

**Figure 12:**
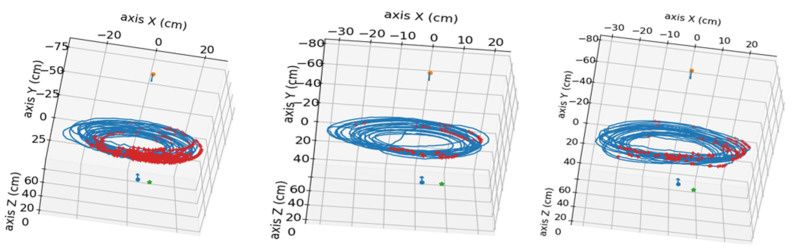
Visualization of the obtained 3D points in the analyzed videos, represented in a blue color. The points with a reprojection error greater than 10 px are marked in red. Red and blue arrows indicate the camera’s position in the scene, and the green dots mark the position of the pot’s base. **Left**: video pair 4 without pole. **Middle**: video pair 7 without pole. **Right**: video pair 10 without pole.

Regarding the reprojection errors, the graphs shown in Figure 10 follow a sawtooth pattern across all processed videos, with the maximum reprojection errors aligned in both the top and side view videos of each time-lapse. Figure 12 shows the positions where the reprojection error is greater than 10 px.

The average reprojection error for each of the analyzed videos is shown in Table 4.

In general, it can be considered that the proposed method is able to achieve excellent results, obtaining an average reprojection error of only 3.7 px, which is below 0.3% of the width of the images. This value can be roughly translated into an estimated average error in the 3D localization of about 0.5 cm, although this measurement depends on other factors, such as the position of the plant tip. The total number of frames where there is a high error (greater than 10 px) is less than 5.4%. On the other hand, although the method required manual correction by an operator during some frames, as we have seen, this only occurred in 8.3% of the frames. In the remaining 91.7% of the frames, the automatic algorithm worked to locate the tip of the plants. Thus, the accuracy and robustness obtained by the proposed method in tracking the plant tips is suitable for practical use in circumnutation movement experimentation.

The direct comparison of our results with other methods is not feasible, since other works measure their results in terms of different parameters. For example, in the “Circumnutation Tracker” software of Stolarz et al. [11], manual annotation of the positions is required for all the frames of the videos. Tenzer and Clifford’s [12] plant monitoring method only segments the plants, without locating the 3D position of the tip, so the accuracy is given in terms of the area under the ROC curve for the classification problem. In the inertial tracker proposed by Geldhof et al. [15], the errors are expressed as the precision of pitch and roll angles, being under 0.5°. The recent method by Mao et al. [13] using deep learning is able to achieve an average error of 1.02 mm in the location of the apex of the plant, in videos of 640 × 480 px of resolution; however, only one video is analyzed per plant, so the 3D locations are not extracted.

**Figure 13:**
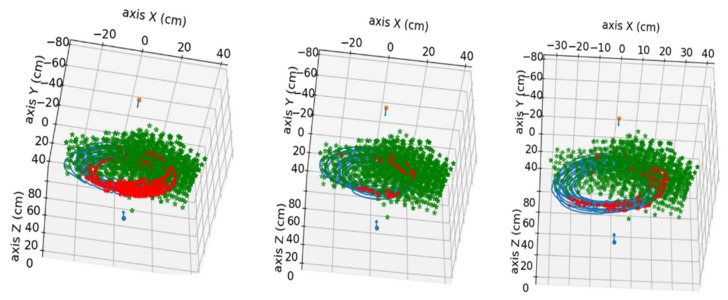
Visualization of 3D points with a reprojection error greater than 10 px (marked in red) and calibrations points (marked in green). **Left**: video pair 4 without pole. **Middle**: video pair 7 without pole. **Right**: video pair 10 without pole. Red and blue arrows indicate the camera’s position in the scene, and the green dots mark the position of the pot’s base.

The largest reprojection errors in our method are found in the area closest to the lateral camera. By overlaying the points used in the calibration onto the trace corresponding to the nutation movement of the plant tip, it is observed that not all the space travelled by the plant tip was covered. Furthermore, the greatest reprojection errors are located in the area where there are no calibration points, as shown in Figure 13.

To prevent this error in future experiments, it will be verified during the calibration process that there are enough calibration points across the entire space traveled by the plant tip in its nutation movement.

## 4. Conclusions

The study of the nutation movement of plants has aroused great interest in the research community in different areas, from philosophy to computer science, and has been a great source of inspiration for new algorithms and bioinspired systems. This paper has presented the development of a stereo vision system for studying the movement of climbing plants. The system consists of a pair of RGB cameras that synchronously record a side and top view of the plants in time-lapse. This study is focused on the common bean as a typical climbing plant model. Subsequently, the images are analyzed with a computer vision algorithm that obtains the tip of the plant and, using a previous calibration of the stereo pair, estimates the position in 3D coordinates. The method is able to extract the correct tip position in 86–98% of cases, depending on the video, with an average reprojection error below 4 px, which is translated to an approximate error in the 3D localization of about 0.5 cm. The proposed method allows researchers to know precisely and robustly the nutation movements of the plants and to compare their behavior under different situations, such as the use or absence of support structures for climbing.

In future work, it would be interesting to apply the latest deep learning methods to perform the accurate segmentation of the plants in the images, as well as subsequent matching between the stereo pair images for a complete estimation of the 3D position of the plant. In this way, it would be possible to know not only the position of the tip of the plant, but also of other parts such as the stems and leaves.

## Figures and Tables

**Table 1:** Technical specifications of the cameras used in the stereo system.

	Characteristics
Sensor	Type: 1/3″ HDR sensor
	Dynamic range: 115 db
	Resolution: 1.3 Mega pixel (1280 × 720 px)
	Pixel size: 4.2 µm
	Sensibility: 3650 mV/lux-sec
Optical lens	Type: CS Mount
	Aperture: F 2.0 Field of view: 112°
	Focal length: 19 mm

**Table 2:** Results of the stereo system calibration. The mean reprojection error (Mean RE) and the standard deviation of the reprojection error (Stdev RE) are shown for the top camera, the side camera, and the complete stereo system.

Reprojection Errors (in Pixels)						
Scenario	Mean RE Top Camera	Stdev RE Top Camera	Mean RE Side Camera	Stdev RE Side Camera	Mean RE Stereo System	Stdev RE Stereo System
No Pole	0.47202	0.34563	0.29831	0.27472	0.61930	0.55591
Pole	0.30597	0.20053	0.28490	0.18405	0.91065	0.80017

**Table 3:** Results of the plant tip detection procedure in the images for the three video pairs. For each video pair, the condition (with or without a supporting pole), the frames that were analyzed, the camera (top or lateral), and the extraction of the tip location (automatic, manual, estimated or interpolated location) are indicated.

			Type of Detection				
Video	Pole	Frames ^1^	View	Automatic	Manual	Estimated	Interpolated
Pair 4	No	2483–4746	Lateral	1586	331	293	54
			Top	1878	74	265	47
Pair 7	Yes	3850–5798	Lateral	1607	272	246	100
			Top	2063	27	113	22
Pair 7	No	750–2600	Lateral	1285	208	287	71
			Top	1724	53	74	0
Pair 10	No	3850–5798	Lateral	1353	251	236	109
			Top	1570	151	209	19

^1^ Range of frames that have been processed from the video.

**Table 4:** Results of the reprojection errors for the three video pairs. The mean reprojection error (Mean RE), the standard deviation of the reprojection error (Stdev RE), the total number of points analyzed, and the number of points with a reprojection error greater than 10 px (RE Points > 10 px) are shown for each video pair.

Reprojection Errors (in Pixels)					
Video	View	Mean RE	Stdev RE	Total Points	RE Points > 10 px
Pair 4	Lateral	3.98679	2.84289	2264	39
	Top	6.49004	5.74389	2264	373
Pair 7	Lateral	2.05453	2.52104	1851	33
	Top	2.66366	3.13255	1851	47
Pair 10	Lateral	2.99391	2.62791	1949	41
	Top	4.08886	3.31717	1949	119

## Data Availability

Data used in this study are available from the authors upon reasonable request.
